# Gender-Specific Longitudinal Association of Sleep Duration with Blood Pressure among Children: Evidence from CHNS 2004–2015

**DOI:** 10.1155/2020/5475297

**Published:** 2020-07-12

**Authors:** Lili Huang, Jiajun Lyu, Zichong Long, Yuanqing Xia, Yiting Chen, Xiuxia Ye, Shenghui Li

**Affiliations:** ^1^School of Public Health, Shanghai Jiao Tong University School of Medicine, Shanghai, China; ^2^Shanghai Children's Medical Center, Shanghai Jiao Tong University School of Medicine, Shanghai, China; ^3^MOE-Shanghai Key Laboratory of Children's Environmental Health, Shanghai Jiao Tong University School of Medicine, Shanghai, China

## Abstract

**Purpose:**

We conducted this study to add the evidence regarding the gender-specific association between sleep duration and blood pressure (BP) in children.

**Methods:**

A secondary analysis was performed among 1000 children aged 7–13 years, who had at least two rounds of survey records in China Health and Nutrition Survey through 2004–2015. Generalized estimating equation was used to explore the gender-specific association of sleep duration with BP. The subgroup analysis was applied in those participants with normal weight.

**Results:**

The time trend of decreasing sleep duration, along with increasing BP level, was observed in each age group during the survey period. Short sleepers (<9 hours per day) have higher level of both systolic BP (SBP) and diastolic BP (DBP) than long sleepers in girls (all *p* < 0.05). By contrast, only SBP was higher in short sleepers among boys (*p* < 0.05). There was gender difference in the association between sleep duration and DBP (*p* for interaction <0.05). The stratification analysis showed that short sleep duration could consistently predict a higher level of diastolic BP (DBP) in both crude (*β* = 2.968, 95% CI: 1.629, 4.306) and adjusted models (*β* = 1.844, 95% CI: 0.273, 3.416) only in girls. Sleep duration was also analyzed as continuous variable, and the very similar associations were observed. Moreover, the established associations can be verified among children with normal weight.

**Conclusions:**

There was a time trend of decreasing sleep duration alongside increasing BP among children from 2004 to 2015. Short sleep duration was independently associated with increased DBP; however, only girls were susceptible to the association.

## 1. Introduction

Pediatric hypertension, an important event that can induce subsequent cardiovascular diseases in childhood and adulthood, has been a public concern during the past several decades [1–3]. Findings from a systematic review and meta-analysis demonstrated that the prevalence of childhood hypertension has been increasing during the past two decades, with a relative increasing rate of 75% to 79% from 2000 to 2015 [4]. In a parallel manner, childhood prehypertension also showed an increasing trend, with a pooled prevalence estimated to be 9.67% (95% CI, 7.26%–12.38%) [4]. Accumulating studies have suggested that prehypertension was implicated in subsequent organ damages, such as left ventricular hypertrophy, carotid intima-media thickness, diastolic dysfunction, and arterial stiffness [5].

Apart from that a number of factors, such as obesity [6], long sedentary time [7], smoking [8], and drinking alcohol [9], have been identified to be associated with blood pressure (BP), sleep has become a focus in recent years. Evidence confirmed that sleep deprivation could induce elevated BP via increasing the activity of sympathetic nervous system and renin-angiotensin system, and as a consequence, more concentration of vasoconstrictor endothelin in blood [10]. The association of insufficient sleep duration with elevated BP has been numerously reported in adults [11–13]. However, as sleep deprivation was more prevalent among children than ever before [14, 15], fewer studies gave attention to its impact on childhood BP.

Although there are several reports to discuss the association of insufficient sleep duration with BP among children and adolescents, most studies were cross-sectional or based on single-center data, and the results were inconsistent and controversial [16–19]. To the best of our knowledge, to date four studies, two in adolescents and two in preschool children, observed the longitudinal association of sleep duration with childhood BP using prospective design [20–23]. A cohort conducted in Tucson, the US, among 334 adolescents reported that total sleep duration at baseline was negatively associated with systolic BP (SBP), but not diastolic BP (DBP) at follow-up 5 years later [20]. Another cohort study in Porto, Portugal, which recruited 1403 adolescents, found that sleep duration at 13 years old was negatively associated with SBP at 17 years old only in boys [21]. By contrast, two newly published data examined the association among relatively younger children [22, 23]. The first one among 5656 European children aged 2–2.99 years demonstrated that short sleep duration (≤9 hours per night) in early childhood could predict both higher SBP and DBP over 2-year follow-up [22]. The other was conducted among 1248 Spanish children, which found that sleep duration at 4 years old was inversely associated with SBP *z* score at 7 years old [23]. Put these findings together, there should be a causal association between short sleep duration and higher BP in childhood; however, the existing contradiction is calling for more evidence. Considering children are undergoing changing development, data from school-aged children is extremely necessary to enrich the evidence. In addition, gender difference has been well known in the prevalence of childhood hypertension [24, 25], while the gender-specific analysis by the same risk exposure has rarely been performed. The limited data revealed the possibility of gender susceptibility in the association of sleep duration with childhood BP [21, 26], and only one longitudinal study explored the topic among late adolescents [21]. Furthermore, obesity is a key confounding factor in the association [20], and the validation in children with normal weight would strengthen the argument.

In the present study, based on the longitudinal surveys from 9 provinces in China, namely, China Health and Nutrition Survey (CHNS), we attempted to explore (a) the shift of sleep duration and blood pressure among children aged 7–13 years old; (b) how sleep duration impacts blood pressure, including SBP, DBP, and elevated BP, defined as hypertension and/or prehypertension; (c) if there is gender-specific association between sleep duration and blood pressure. To clearly clarify the association, the analysis was also applied in subgroup without overweight/obesity.

## 2. Methods

### 2.1. Participants and Study Design

This study was designed based on a subset from CHNS, which is an ongoing household-based longitudinal survey covering 9 provinces across China (Heilongjiang, Liaoning, Shandong, Henan, Jiangsu, Hunan, Hubei, Guangxi, and Guizhou) from 1989 to the present. Stratified probability sampling method was used to recruit the study population [27]. Each wave measured dietary, anthropometric parameters, and clinical detection. Information regarding sleep duration was collected starting from 2004. This study was approved by the institutional review committees of the University of North Carolina at Chapel Hill, the National Institute of Nutrition and Food Safety, and the Chinese Center for Disease Control and Prevention. Each participant signed an informed consent by their parents or guardians.

Participants enrolled in the present study were children aged 7–13 years, deriving among 4824 records from surveys at 2004, 2006, 2009, 2011, and 2015. Participants with missing data on sleep duration or BP measurements were excluded. In addition, only those children who participated in two or more survey waves were included, which referred to several CHNS-based studies and adopted the same participant inclusion strategy [28, 29]. The final sample consisted of 1000 participants with 2191 records. Among 1000 participants, 812 have two records, 185 have three records, and 3 have four records (Figure S1).

### 2.2. Measurements of Sleep Duration and BP

Sleep duration was evaluated by a question “How many hours each day do you usually sleep, including daytime and nighttime (hours)?,” which was reported by participants themselves (≥12 years old) or by their parents or guardians (<12 years old). According to consensus statement of the American Academy of Sleep Medicine, <9 hours per day was defined as insufficient sleep duration for children aged 6–12 years old [30], and based on this, sleep duration was categorized into two groups as <9 hours per day vs. ≥9 hours per day.

The measurements of BP were performed by trained physicians following standardized protocols from the World Health Organization. The participants were asked to sit for ten minutes before BP measurement. Seated SBP/DBP was measured in triplicate with 10-minute rest interval between the measurements, using mercury manometers. The three readings were averaged. According to the updated recommendations of the fourth report of the National High Blood Pressure Education Program Working Group on Children and Adolescents [31], SBP or DBP ≥90th percentiles and <95th percentiles was defined as prehypertension, SBP or DBP ≥95th percentiles was defined as hypertension, based on specific gender, age, and height. Due to the relatively small proportion of hypertension in our study sample, prehypertension and hypertension were converged together as elevated BP.

### 2.3. Covariates

BMI (body mass index, weight (kg) divided by the square of height (m)) *z* score and waist circumference were used to assess overweight and obesity status. BMI *z* score was calculated using the followed formula: (individual BMI value–mean BMI value)/BMI standard deviation (SD), based on specific gender and age published by WHO [32]. 1≤ individual BMI *z* score <2 was defined as overweight, and individual BMI *z* score ≥2 was defined as obesity. We converged overweight and obesity into one group since only small proportion of overweight and obesity was identified in our study sample.

The frequency of physical activities per week was used to evaluate the level of physical exercise, which was grouped into three categories: ≤2 times per week; 3–7 times per week; and ≥8 times per week. We summarized the major types of screen exposure to evaluate average screen time per day, including watching television; video compact disc, video games, surfing the internet, joining chat rooms, and playing computer games. Average screen time was grouped into three categories: <1 hours per day, 1-2 hours per day, and ≥2 hours per day.

Regions were grouped into four categories: northeastern area (Liaoning, Heilongjiang), east coast (Beijing, Jiangsu, Shandong, and Shanghai), central area (Henan, Hubei, and Hunan), and western area (Chongqing, Guangxi, and Guizhou). Per capita household income was classified into tertiles. Urbanization index was calculated using 12 components, which has been validated in previous studies [33], and the index was categorized into year-specific tertiles. Parental hypertension history (yes or no) and the year of study entry were also considered.

### 2.4. Statistical Analysis

Descriptive analysis was performed based on survey time, expressed as mean (SD) or number (percentage). ANOVA, Kruskal–Wallis test, *t*-test, and chi-squared test were conducted to exam the group difference where appropriate.

Sleep duration was analyzed as both continuous and categorical (<9 hours and ≥9 hours) form. BP was described as three forms: SBP and DBP were continuous variables, and elevated BP was dichotomous variable (yes or no). Generalized estimating equation was used to explore the longitudinal association between sleep duration and BP. In crude models, only sleep duration was entered to analyze the relationship. Then, we adjusted for age, gender, and parents' hypertension history in adjusted model 1. In the adjusted model 2, waist circumference, BMI *z* score, frequency of physical activities per week, screen time per week, per capita household income, regions, urbanization index, and year of study entry were additionally adjusted. In addition, the interaction effect of sleep duration with other characteristics of participants, such as gender, BMI *z* score, waist circumference, and frequency of physical activities, were intended to be explored. The product term of sleep duration with gender, BMI *z* score, waist circumference, and frequency of physical activities were, respectively, applied to examine the possible interaction effect when examining the association between sleep duration and BP. The interaction between gender and sleep duration was significant; therefore, our analyses were stratified by gender to explore gender-specific association. In the subgroup analysis, we check the relationship among children with normal weight.

Data analysis was performed by IBM SPSS Statistics (version 24.0, IBM Corp.) and *R* (version 3.6.0). *p* < 0.05 (two-tailed) was regarded as statistically significant.

## 3. Results

### 3.1. Participants' Characteristics over Time

Participants included in this analysis were aged 9.05 (SD = 1.47) to 9.84 (SD = 2.07) years old over the survey years from 2004 to 2011; however, 11.82 (SD = 0.82) years in 2015 were slightly older (Table 1). The proportion of girls was nearly a half at each survey. The prevalence of sleep duration <9 hours was increased over time, from 19.0% in 2004 to 56.0% in 2015 (*p* < 0.001). The level of SBP and DBP was increased over time, as well as the prevalence of elevated BP (all *p* < 0.05). These trends were similarly observed in both males and females (Tables S1-S2).

### 3.2. Distribution of Blood Pressure Based on Sleep Duration

**Figure 1:**
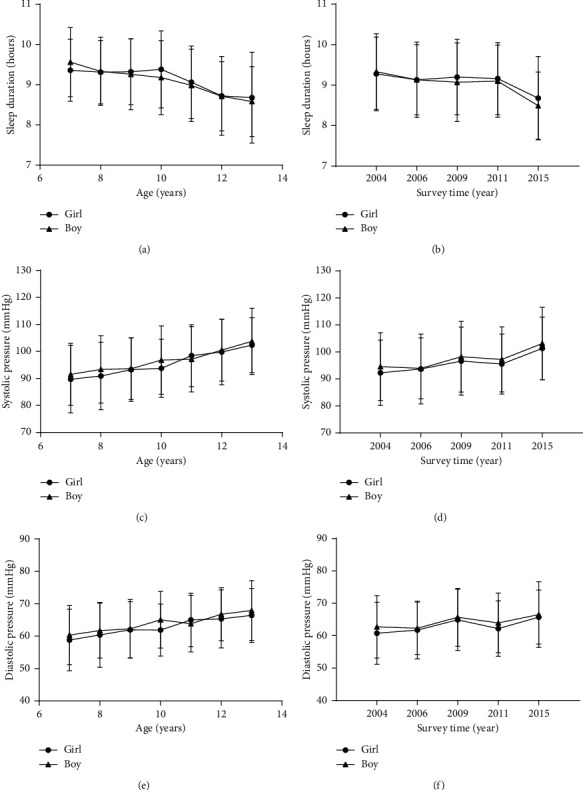
The trend of sleep duration and BP level (mean and error) with increasing age (a) and over survey time 2004–2015 (b).

In both genders, sleep duration was decreased with age and survey time; meanwhile, SBP and DBP levels were increased with age and survey time (Figure 1). Compared to girls, boys have higher SBP (96.70 mmHg vs. 95.32 mmHg, *p* = 0.01), higher DBP (64.00 mmHg vs. 62.80 mmHg, *p* = 0.002) level, and correspondingly, higher prevalence of elevated BP (*p* = 0.03) (Table 2). As shown in Table 3, the mean levels of both SBP (98.42 mmHg vs. 94.25 mmHg, *p* < 0.001) and DBP (65.10 mmHg vs. 62.01 mmHg, *p* < 0.001) were significantly higher among those slept <9 hours than those slept ≥9 hours in girls. Correspondingly, the proportions of elevated BP were higher in the group sleep duration <9 hours per day than those in the group sleep duration ≥9 hours (15.8% vs. 10.5%, *p* < 0.05). By contrast to girls, the difference in DBP level and the prevalence of elevated BP were not found between sleep duration <9 hours and sleep duration ≥9 hours among boys.

### 3.3. Association of Sleep Duration with BP

It can be seen in Table S3, the effect of gender on the association between sleep duration and DBP was significant (*p* for interaction <0.05). We, therefore, performed gender-stratified analysis to examine the association between sleep duration and BP (Table 4). Except for elevated BP in boys, the associations were generally established in both genders. However, the estimated effect of short sleep duration, compared to sleep duration ≥9 hours, with SBP and DBP was larger in girls (*β* = 4.014, 95% CI: 2.237, 5.792; *β* = 2.968, 95% CI: 1.629, 4.306, respectively) than in boys (*β* = 3.586, 95% CI: 2.030, 5.142; *β* = 1.238, 95% CI: 0.092, 2.384, respectively). After adjusted for age and parents' hypertension history, the effect of short sleep duration on DBP was kept statistically significant only in girls but somewhat attenuated (*β* = 1.850, 95% CI: 0.428, 3.272). We further adjusted lifestyles and social factors in model 2; the associated effect of short sleep duration on DBP in girls was still significant, in which short sleep duration could increase 1.844 (0.273, 3.416) mmHg of DBP level compared with sleep duration ≥9 hours. When sleep duration was analyzed as continuous variable, the similar associations were observed. The negative association of sleep duration with DBP (*β* = −1.121, 95% CI: −1.869, −0.373) was still significant only in girls after adjusted covariates. In subgroup analysis, we excluded participant with overweight and/or obesity to check the associations between sleep duration and BP in normal-weight participants (*n* = 794 with 1717 records). The results were largely repeatable and some estimated effects became prominent compared with analyses in all sampled participants (Table 4). Considering the relatively large variability of characteristics in 2015, we repeated the analysis by excluding participants in 2015, where the results were largely unchanged (Table S4).

## 4. Discussion

In the present study, we are the first to observe time trend of sleep duration and BP level among Chinese children aged 7–13 years old through a longitudinal survey. Moreover, the gender-specific associations between sleep duration and BP status were particularly examined. Our findings demonstrated a tendency of increasing SBP, DBP, and elevated BP alongside the declining sleep duration through 2004 to 2015. Short sleep duration, defined as **<**9 hours per day, was significantly associated with an increased risk of higher DBP. However, this effect showed a gender difference that only girls were susceptible to the association. We also applied subgroup analysis among children with normal weight to confirm the association; the similar and strengthened results further enforced the evidence.

The prevalence of hypertension and prehypertension among children is increasing worldwide. A systematic review estimated the global prevalence of hypertension from 2000 to 2015, where it was shown that the increasing rate of hypertension reached as high as 75% to 79% among participants aged 6–19 years [4]. Similarly, our study also suggested an increased trend of elevated BP (13.9% in 2004 and 15.3% in 2015). Generally, the prevalence of elevated BP rate in our sampled children was similar to their peers in India (prehypertension: 8.25% and hypertension: 3.03%) [34] and Brazil (prehypertension: 8.75% and hypertension: 3.21%) [35]. However, data from Mexico showed much higher prevalence of prehypertension (11.97%) and hypertension (6.18%) among children aged 5–11 years old [36]. By contrast, as low as 2.5% of prehypertension was reported among children aged 3–17 years old from Nigeria [37].

The association of short sleep duration with increased odd ratio of hypertension among children has been reported in other studies [18, 22]. Four prospective studies have reported the longitudinal association of sleep duration with blood pressure [20–23], as described in the Introduction. Consistent with these findings, our study also found that short sleep duration could increase the risk of elevated BP, however, only in girls, especially for DBP. Contrary to our findings, several previous studies found the gender-specific association only in boys. The longitudinal data from Porto adolescents reported sleep duration at 13 years old was inversely associated with SBP at 17 years old only in boys [21]. A previous study among Chinese children aged 11–14 years also found that short sleep duration was associated with hypertension only in boys; however, the data was cross-sectionally collected [26]. Our data is the first to focus on the longitudinal association among Chinese children. Age differences among studies may be partly responsible for controversial findings. During puberty, the biological development in females is earlier than males, which makes sleep needs change a lot between genders [38, 39]. It has been proposed that females may need more sleep duration during prepuberty and early puberty while males need longer sleep at late puberty. The gender difference should be emphasized in analyzing the association between sleep duration and childhood BP.

The mechanism to explain such gender-specific effect was still unclear. Environmental and/or biological (gender hormone) factors may be partly responsible for such gender-specific effect [40]. This gender-specific effect also frequently occurred in adults [41–44]. A cross-sectional study in Chinese adults found that high BP was associated with short sleep duration among women, whereas high BP was associated with long sleep duration among men [43]. A recent prospective cohort among 4,184 adults also revealed a gender-divergent response in the association of sleep parameters with subclinical atherosclerosis [44]. In addition to sleep requirement by gender, culture, anxiety, and depressive as well as other physical and psychological factors also should be considered [45, 46]. Currently, more biological mechanism exploration is necessary to understand the gender-specific sleep characteristic and its health effects.

The present study has several strengths. Firstly, we used several years data from 2004 to 2015 to explore the time trend of seep duration and BP values. Secondly, in the association between sleep duration and BP, we adjusted important confounders, including parents' hypertension history, social environment, lifestyles, and BMI *z* score. Thirdly, we particularly explored the possible gender difference. Moreover, the results were validated in subgroup with normal weight, which further enforced the evidence. However, there were also some limitations that should be noted. Firstly, the measurements of sleep duration were self- or parents-reported; measurements (such as actigraphy) are more objective. However, reported sleep duration was particularly suitable in large epidemiology studies for its good feasibility and cost-effective features. Fortunately, a previous study has confirmed a moderate correlation between objective and subjective measurements [47]. Secondly, the questionnaire of this survey did not characterize sleep behaviors in detail; sleep parameters such as daytime naps, sleep quality, and bed and wake time were unavailable.

## 5. Conclusion

According to our findings, the time trend of decreasing sleep duration while increasing BP in children aged 7–13 years old over time from 2004 to 2015 was found. A gender-specific association was identified; that is, girls were more susceptible to get higher blood pressure when they are exposed to short sleep duration. The association could be verified by sensitive analyses in subgroup children with normal weight. The finding still needs more evidence to confirm; however, it should be cautious to take sleep evaluation into consideration in childhood hypertension intervention.

## Figures and Tables

**Table 1:** Sample characteristics (mean (SD) or *n* (percentage)) over survey years from 2004 to 2015.

	2004 (*n* = 374)	2006 (*n* = 566)	2009 (*n* = 530)	2011 (*n* = 522)	2015 (*n* = 209)	Time trend (*p*)
Participants' personal characteristics						
Age (years)	9.05 (1.47)	9.92 (1.95)	10.12 (1.90)	9.84 (2.07)	11.82 (0.82)	<0.001
Gender						
Boy	196 (52.4)	305 (54.7)	296 (55.8)	278 (53.3)	107 (51.2)	0.739
Girl	178 (47.6)	252 (45.3)	234 (44.2)	244 (46.7)	102 (48.8)	
Waist circumference	57.41 (6.90)	58.47 (8.12)	60.64 (10.04)	60.67 (11.21)	65.42 (12.36)	<0.001
BMI *z* score	−0.08 (0.82)	−0.02 (0.93)	−0.04 (0.96)	0.05 (1.09)	0.20 (1.24)	0.099
Physical activities frequency per week (times)						
<3	156 (47.4)	166 (28.8)	103 (26.0)	110 (29.1)	124 (42.1)	<0.001
3–7	136 (41.3)	158 (42.1)	184 (46.5)	183 (48.4)	72 (40.4)	
≥8	37 (11.2)	109 (29.1)	109 (27.5)	85 (22.5)	31 (17.4)	
Screen time per day (hours)						
<1	123 (35.8)	155 (29.6)	125 (24.1)	206 (24.0)	55 (28.5)	<0.001
1-2	134 (39.0)	187 (35.7)	196 (37.8)	202 (40.1)	54 (28.0)	
≥3	87 (25.3)	182 (34.7)	197 (38.0)	181 (30.9)	84 (43.5)	
Family and community information						
Parents' hypertension history						
Yes	9 (2.6)	13 (2.5)	24 (5.0)	24 (5.6)	14 (8.0)	0.006
No	342 (97.4)	499 (97.5)	459 (95.0)	425 (94.4)	161 (92.0)	
Per capita house income (yuan)						
Tertiles 1	165 (44.6)	356 (43.0)	147 (28.2)	187 (24.4)	42 (23.3)	<0.001
Tertiles 2	131 (35.4)	207 (38.1)	192 (36.9)	167 (32.6)	69 (20.8)	
Tertiles 3	71 (20.0)	103 (18.9)	182 (34.9)	220 (43.0)	122 (58.9)	
Urbanization index						
Tertiles 1	122 (32.6)	203 (36.5)	201 (37.9)	197 (37.7)	84 (40.2)	0.711
Tertiles 2	138 (36.9)	186 (33.5)	175 (33.0)	169 (32.4)	71 (34.0)	
Tertiles 3	114 (30.5)	167 (30.0)	154 (29.1)	156 (29.9)	54 (25.8)	
Region						
Central	90 (24.1)	131 (23.6)	134 (25.3)	128 (24.5)	66 (20.1)	<0.001
East coast	61 (16.3)	95 (17.1)	98 (18.5)	106 (20.3)	59 (28.2)	
Northeastern	98 (26.2)	120 (21.6)	73 (13.8)	52 (10.0)	18 (9.0)	
Western	125 (33.4)	210 (37.8)	225 (42.5)	236 (45.2)	90 (41.0)	
Sleep duration and blood pressure						
Sleep duration (hours)	9.30 (0.92)	9.13 (0.90)	9.13 (0.95)	9.13 (0.90)	8.58 (0.93)	<0.001
Sleep duration (hours)						
<9	71 (19.0)	136 (24.5)	135 (25.5)	128 (24.5)	117 (56.0)	<0.001
≥9	303 (81.0)	420 (75.5)	395 (74.5)	394 (75.5)	92 (44.0)	
SBP (mmHg)	93.48 (12.38)	93.80 (12.03)	97.50 (12.93)	96.42 (11.67)	102.24 (12.59)	<0.001
DBP (mmHg)	61.79 (9.66)	62.04 (8.48)	65.35 (9.17)	63.12 (8.95)	66.14 (9.30)	<0.001
Elevated BP						
Yes	52 (13.9)	53 (9.5)	95 (17.9)	66 (12.6)	32 (15.3)	0.002
No	322 (86.1)	503 (90.5)	435 (82.1)	456 (87.4)	177 (84.7)	

BMI: body mass index; SBP: systolic blood pressure; DBP: diastolic blood pressure; BP: blood pressure.

**Table 2:** The distribution of BP and sleep duration stratified by gender, converging all survey times.

	Boy (*n* = 1181)	Girl (*n* = 1010)	*t*/*χ*^2^	*p*
Sleep duration				
Continuous	9.08 (0.93)	9.14 (0.94)	−1.31	0.190
Category				
<9 h	327 (27.7)	260 (25.7)	1.05	0.305
≥9 h	854 (72.3)	750 (74.3)		
Blood pressure				
SBP	96.70 (12.63)	95.32 (12.40)	2.57	0.010
DBP	64.00 (9.15)	62.80 (9.18)	3.04	0.002
Elevated BP				
Yes	178 (15.1)	120 (11.9)	4.72	0.030
No	1003 (84.9)	890 (88.1)		

**Table 3:** The distribution of BP based on sleep duration, converging all survey times.

		SBP			DBP			Elevated BP		
Overall	*n*	Mean (SD)	*t*	*p*	Mean (SD)	*t*	*p*	*N* (%)	*χ* ^2^	*p*
<9 h	587	98.83 (13.00)	6.104	<0.001	64.84 (9.29)	4.307	<0.001	98 (16.7)	6.532	0.011
≥9 h	1604	95.05 (12.22)			62.94 (9.09)			200 (12.5)		
Boy										
<9 h	327	99.15 (13.20)	4.149	<0.001	64.63 (9.33)	1.473	0.141	57 (17.4)	1.966	0.161
≥9 h	854	95.77 (12.28)			63.76 (9.07)			121 (14.2)		
Girl										
<9 h	260	98.42 (12.46)	4.715	<0.001	65.10 (9.26)	4.725	<0.001	41 (15.8)	5.056	0.025
≥9 h	750	94.25 (12.10)			62.01 (9.03)			79 (10.5)		

**Table 4:** The association of sleep duration with SBP, DBP, and elevated BP stratified by gender in 2004–2015.

	All participants			Normal weight group		
	SBP	DBP	Elevated BP	SBP	DBP	Elevated BP
	*β* (95% CI)	*β* (95% CI)	OR (95% CI)	*β* (95% CI)	*β* (95% CI)	OR (95% CI)
*Boy*	*n* = 1181			*n* = 921		
Crude model						
Continuous	**−2.145 (−2.936, −1.355)**	**−0.893 (−1.442, −0.344)**	**0.824 (0.695, 0.977)**	**−2.429 (−3.313, −1.546)**	**−1.015 (−1.632, −0.398)**	**0.738 (0.605, 0.900)**
<9 h	**3.586 (2.030, 5.142)**	**1.238 (0.092, 2.384)**	1.282 (0.916, 1.794)	**3.813 (1.360, 5.490)**	1.141 (−0.150, 2.433)	1.480 (0.995, 2.199)
≥9 h	Reference	Reference	Reference	Reference	Reference	Reference
Adjusted model^1^						
Continuous	−0.728 (−1.608, 0.151)	0.178 (−0.409, 0.764)	0.857 (0.703, 1.044)	**−1.070 (−2.058, −0.083)**	−0.013 (−0.673, 0.647)	**0.766 (0.610, 0.961)**
<9 h	0.964 (−0.749, 2.676)	−0.614 (−1.782, 0.554)	1.199 (0.817, 1.759)	1.225 (−0.693, 3.143)	−0.674 (−2.010, 0.662)	1.381 (0.877, 2.175)
≥9 h	Reference	Reference	Reference	Reference	Reference	Reference
Adjusted model^2^						
Continuous	−0.628 (−1.709, 0.453)	0.541 (−0.190, 1.272)	0.948 (0.741, 1.212)	−0.645 (−1.853, 0.563)	0.504 (−0.306, 1.314)	0.923 (0.703, 1.212)
<9 h	0.500 (−1.526, 2.525)	−1.251 (−2.656, 0.154)	1.002 (0.618, 1.627)	0.536 (−1.747, 2.818)	−1.350 (−2.924, 0.225)	1.050 (0.607, 1.819)
≥9 h	Reference	Reference	Reference	Reference	Reference	Reference
*Girl*	*n* = 1010			*n* = 796		
Crude model						
Continuous	**−1.943 (−2.752, −1.134)**	**−1.503 (−2.088, −0.918)**	0.804 (0.634, 1.019)	**−1.690 (−2.609, −0.772)**	**−1.339 (−2.014, −0.665)**	0.830 (0.619, 1.112)
<9 h	**4.014 (2.237, 5.792)**	**2.968 (1.629, 4.306)**	**1.564 (1.002, 2.441)**	**4.026 (2.019, 6.033)**	**3.195 (1.644, 4.747)**	**1.749 (1.029, 2.974)**
≥9 h	Reference	Reference	Reference	Reference	Reference	Reference
Adjusted model^1^						
Continuous	**−0.954 (−1.806, −0.102)**	**−1.106 (−1.730, −0.483)**	0.780 (0.603, 1.007)	−0.685 (−1.623, 0.254)	**−0.919 (−1.608, −0.231)**	0.828 (0.611, 1.121)
<9 h	1.411 (−0.455, 3.277)	**1.850 (0.428, 3.272)**	1.610 (0.995, 2.607)	1.401 (−0.667, 3.470)	**1.914 (0.311, 3.518)**	1.691 (0.958, 2.985)
≥9 h	Reference	Reference	Reference	Reference	Reference	Reference
Adjusted model^2^						
Continuous	−0.650 (−1.711, 0.411)	**−1.121 (−1.869, −0.373)**	0.810 (0.577, 1.136)	−0.527 (−1.702, 0.649)	**−1.094 (−1.902, −0.286)**	0.845 (0.569, 1.255)
<9 h	1.144 (−1.073, 3.362)	**1.844 (0.273, 3.416)**	1.584 (0.890, 2.817)	1.153 (−1.318, 3.623)	**1.973 (0.207, 3.738)**	1.667 (0.835, 3.327)
≥9 h	Reference	Reference	Reference	Reference	Reference	Reference

OR: odds ratios; CI: confidence interval. Model 1 adjusted for age and parents' hypertension history. Model 2 additionally adjusted for survey time, waist circumference, BMI *z* score, urbanization, per capita household income, region, frequency of physical activities per week, and screen time per day.

## Data Availability

The data used to support the findings of this study are available from the website of China Health and Nutrition Survey (http://www.cpc.unc.edu/projects/china).

## Supplementary Materials

Supplementary Materials
*Table S1*. Sample characteristics [mean (SD) or *n* (percentage)] in boys over survey years from 2004 to 2015. *Table S2*. Sample characteristics [(mean (SD) or *n* (percentage)] in girls over survey years from 2004 to 2015. *Table S3*. The association of sleep duration with BP in 2004–2015. *Table S4*. The association of sleep duration with SBP, DBP, and elevated BP stratified by gender in 2004–2011. *Figure S1*. Sampling flowchart and participants' enrollment.

## References

[1] Dionne J. M. (2017). Updated guideline may improve the recognition and diagnosis of hypertension in children and adolescents; review of the 2017 AAP blood pressure clinical practice guideline. *Current Hypertension Reports*.

[2] Conkar S., Yılmaz E., Hacıkara Ş., Bozabalı S., Mir S. (2015). Is daytime systolic load an important risk factor for target organ damage in pediatric hypertension?. *The Journal of Clinical Hypertension*.

[3] Daniels S. R., Lipman M. J., Burke M. J., Loggie J. M. H. (1991). The prevalence of retinal vascular abnormalities in children and adolescents with essential hypertension. *American Journal of Ophthalmology*.

[4] Song P., Zhang Y., Yu J. (2019). Global prevalence of hypertension in children: a systematic review and meta-analysis. *JAMA Pediatrics*.

[5] Urbina E. M., Khoury P. R., McCoy C., Daniels S. R., Kimball T. R., Dolan L. M. (2011). Cardiac and vascular consequences of pre-hypertension in youth. *The Journal of Clinical Hypertension*.

[6] Oyekale A. S. (2019). Effect of obesity and other risk factors on hypertension among women of reproductive age in Ghana: an instrumental variable probit model. *International Journal of Environmental Research Public Health*.

[7] Lim M. S., Park B., Kong I. G. (2017). Leisure sedentary time is differentially associated with hypertension, diabetes mellitus, and hyperlipidemia depending on occupation. *BMC Public Health*.

[8] Virdis A., Giannarelli C., Neves M. F., Taddei S., Ghiadoni L. (2010). Cigarette smoking and hypertension. *Current Pharmaceutical Design*.

[9] O’Keefe J. H., Bhatti S. K., Bajwa A., DiNicolantonio J. J., Lavie C. J. (2014). Alcohol and cardiovascular health: the dose makes the poison…or the remedy. *Mayo Clinic Proceedings*.

[10] Kato M., Phillips B. G., Sigurdsson G., Narkiewicz K., Pesek C. A., Somers V. K. (2000). Effects of sleep deprivation on neural circulatory control. *Hypertension*.

[11] Gangwisch J. E., Heymsfield S. B., Boden-Albala B. (2006). Short sleep duration as a risk factor for hypertension. *Hypertension*.

[12] Gottlieb D. J., Redline S., Nieto F. J. (2006). Association of usual sleep duration with hypertension: the sleep heart health study. *Sleep*.

[13] Stang A., Moebus S., Möhlenkamp S., Erbel R., Jöckel K. H. (2008). Gender-specific associations of short sleep duration with prevalent hypertension. *Hypertension*.

[14] Garaulet M., Ortega F. B., Ruiz J. R. (2011). Short sleep duration is associated with increased obesity markers in European adolescents: effect of physical activity and dietary habits. The HELENA study. *International Journal of Obesity*.

[15] Wheaton A. G., Jones S. E., Cooper A. C., Croft J. B. (2018). Short sleep duration among middle school and high school students—United States, 2015. *MMWR. Morbidity and Mortality Weekly Report*.

[16] Sampei M., Dakeishi M., Wood D. C., Murata K. (2006). Impact of total sleep duration on blood pressure in preschool children. *Biomedical Research*.

[17] Martikainen S., Pesonen A. K., Feldt K. (2011). Poor sleep and cardiovascular function in children. *Hypertension*.

[18] Kuciene R., Dulskiene V. (2014). Associations of short sleep duration with prehypertension and hypertension among Lithuanian children and adolescents: a cross-sectional study. *BMC Public Health*.

[19] Tsampalieros A., Blinder H., Hoey L. (2019). Obstructive sleep apnea and hypertension in pediatric chronic kidney disease. *Pediatric Nephrology*.

[20] Archbold K. H., Vasquez M. M., Goodwin J. L., Quan S. F. (2012). Effects of sleep patterns and obesity on increases in blood pressure in a 5-year period: report from the tucson children’s assessment of sleep apnea study. *The Journal of Pediatrics*.

[21] Paciência I., Araújo J., Ramos E. (2016). Sleep duration and blood pressure: a longitudinal analysis from early to late adolescence. *Journal of Sleep Research*.

[22] Sparano S., Lauria F., Ahrens W. (2019). Sleep duration and blood pressure in children: analysis of the pan-European IDEFICS cohort. *The Journal of Clinical Hypertension*.

[23] Bawaked R. A., Fernandez-Barres S., Navarrete-Munoz E. M. (2019). Impact of lifestyle behaviors in early childhood on obesity and cardiometabolic risk in children: results from the Spanish INMA birth cohort study. *Pediatrics Obesity*.

[24] Wells J. C. K., Hallal P. C., Reichert F. F., Menezes A. M. B., Araújo C. L. P., Victora C. G. (2008). Sleep patterns and television viewing in relation to obesity and blood pressure: evidence from an adolescent Brazilian birth cohort. *International Journal of Obesity*.

[25] Santos E. S. G., Souza O. F. (2020). Association of sleep duration and blood pressure in adolescents: a multicenter study. *American Journal of Hypertension*.

[26] Guo X., Zheng L., Li Y. (2011). Association between sleep duration and hypertension among Chinese children and adolescents. *Clinical Cardiology*.

[27] Popkin B. M., Du S., Zhai F., Zhang B. (2010). Cohort profile: the China health and nutrition survey—monitoring and understanding socio-economic and health change in China, 1989–2011. *International Journal of Epidemiology*.

[28] Feng X., Liu Q., Li Y., Zhao F., Chang H., Lyu J. (2019). Longitudinal study of the relationship between sleep duration and hypertension in Chinese adult residents (CHNS 2004–2011). *Sleep Medicine*.

[29] Zhao F., Liu Q., Li Y., Feng X., Chang H., Lyu J. (2020). Association between alcohol consumption and hypertension in Chinese adults: findings from the CHNS. *Alcohol*.

[30] Paruthi S., Brooks L. J., D’Ambrosio C. (2016). Recommended amount of sleep for pediatric populations: a consensus statement of the American academy of sleep medicine. *Journal of Clinical Sleep Medicine*.

[31] National High Blood Pressure Education Program Working Group on High Blood Pressure in Children and Adolescents (2004). The fourth report on the diagnosis, evaluation, and treatment of high blood pressure in children and adolescents. *Pediatrics*.

[32] de Onis M., Onyango A. W., Borghi E., Siyam A., Nishida C., Siekmann J. (2007). Development of a WHO growth reference for school-aged children and adolescents. *Bulletin of the World Health Organization*.

[33] Jones-Smith J. C., Popkin B. M. (2010). Understanding community context and adult health changes in China: development of an urbanicity scale. *Social Science & Medicine*.

[34] Patil R., Garg B. (2014). Prevalence of hypertension and variation in blood pressure among school children in rural area of Wardha. *Indian Journal of Public Health*.

[35] Rinaldi A. E. M., Nogueira P. C. K., Riyuzo M. C. (2012). Prevalência de pressão arterial elevada em crianças e adolescentes do ensino fundamental. *Revista Paulista de Pediatria*.

[36] Saury-Paredes L. A. (2016). Prevalence of high blood pressure and their association with body mass index in children between 5 and 11 years of Nahbalam, Yucatan. *Gaceta Medica De Mexico*.

[37] Okpokowuruk F. S., Akpan M. U., Ikpeme E. E. (2017). Prevalence of hypertension and prehypertension among children and adolescents in a semi-urban area of Uyo Metropolis, Nigeria. *The Pan African Medical Journal*.

[38] Sadeh A., Dahl R. E., Shahar G., Rosenblat-Stein S. (2009). Sleep and the transition to adolescence: a longitudinal study. *Sleep*.

[39] Matricciani L. A., Olds T. S., Blunden S., Rigney G., Williams M. T. (2012). Never enough sleep: a brief history of sleep recommendations for children. *Pediatrics*.

[40] Antonijevic I. A., Murck H., Frieboes R.-M., Holsboer F., Steiger A. (1999). On the gender differences in sleep-endocrine regulation in young normal humans. *Neuroendocrinology*.

[41] Kabrita C., Hajjar-Muça T. (2016). Sex-specific sleep patterns among university students in Lebanon: impact on depression and academic performance. *Nature and Science of Sleep*.

[42] Doi Y., Minowa M., Uchiyama M., Okawa M. (2001). Subjective sleep quality and sleep problems in the general Japanese adult population. *Psychiatry and Clinical Neurosciences*.

[43] Wang H., Zee P., Reid K. (2011). Gender-specific association of sleep duration with blood pressure in rural Chinese adults. *Sleep Medicine*.

[44] Domínguez F., Fuster V., Fernández-Alvira J. M. (2019). Association of sleep duration and quality with subclinical atherosclerosis. *Journal of The American College of Cardiology*.

[45] Handa R. J., Burgess L. H., Kerr J. E., O’Keefe J. A. (1994). Gonadal steroid hormone receptors and sex differences in the hypothalamo-pituitary-adrenal axis. *Hormones and Behavior*.

[46] Conklin A. I., Yao C. A., Richardson C. G. (2018). Chronic sleep deprivation and gender-specific risk of depression in adolescents: a prospective population-based study. *BMC Public Health*.

[47] Lauderdale D. S., Knutson K. L., Yan L. L., Liu K., Rathouz P. J. (2008). Self-reported and measured sleep duration. *Epidemiology*.

