# Supplementary material for: Gender-Specific Longitudinal Association of Sleep Duration with Blood Pressure among Children: Evidence from CHNS 2004–2015
Source: Int J Hypertens. 2020 Jul 12;2020:5475297. doi: 10.1155/2020/5475297 (PMC7374217; doi:10.1155/2020/5475297)
Supplement: Supplementary Materials — Table S1. Sample characteristics [mean (SD) or n (percentage)] in boys over survey years from 2004 to 2015. Table S2. Sample characteristics [(mean (SD) or n (percentage)] in girls over survey years from 2004 to 2015. Table S3. The association of sleep duration with BP in 2004–2015. Table S4. The association of sleep duration with SBP, DBP, and elevated BP stratified by gender in 2004–2011. Figure S1. Sampling flowchart and participants' enrollment. [file 5475297.f1.docx]

**Table S1** Sample characteristics [(mean (SD) or n (percentage)] in boys over survey year from 2004 to 2015

|  | **2004 (n=196)** | **2006 (n=304)** | **2009 (n=296)** | **2011 (n=278)** | **2015 (n=107)** | **Time trend (*p*)** |
| --- | --- | --- | --- | --- | --- | --- |
| **Participants’ personal characteristics** | |  |  |  |  |  |
| Age(years) | 9.11 (1.50) | 9.86 (2.03) | 10.23 (1.92) | 9.95 (2.11) | 11.85 (0.83) | <0.001 |
| BMI z score | -0.07 (0.83) | -0.03 (0.89) | -0.04 (0.99) | 0.06 (1.13) | 0.17 (1.18) | 0.443 |
| Waist circumference | 58.62 (6.71) | 59.57 (8.63) | 62.39 (10.86) | 61.68 (11.21) | 65.69 (13.66) | <0.001 |
| Physical exercise frequency per week | |  |  |  |  |  |
| <3 | 82 (48.2) | 58 (27.9) | 62 (27.6) | 57 (29.8) | 41 (43.6) | <0.001 |
| 3-7 | 70 (41.2) | 83 (39.9) | 108 (48.0) | 89 (46.6) | 33 (35.1) |  |
| ≥8 | 18 (10.6) | 67 (32.2) | 55 (24.4) | 45 (23.6) | 20 (21.3) |  |
| Screen time per day |  |  |  |  |  |  |
| <1h | 61 (32.8) | 78 (26.7) | 65 (22.6) | 65 (24.3) | 24 (23.8) | 0.080 |
| 1-2h | 73 (39.2) | 99 (33.9) | 108 (37.6) | 106 (39.6) | 32 (31.7) |  |
| ≥3h | 52 (28.0) | 115 (39.4) | 114 (39.7) | 97 (36.2) | 45 (44.6) |  |
| **Family and community information** |  |  |  |  |  |  |
| Parents hypertension history |  |  |  |  |  |  |
| No | 182 (97.8) | 272 (97.5) | 262 (96.0) | 230 (93.9) | 81 (90.0) | 0.009 |
| Yes | 4 (2.2) | 7 (2.5) | 11 (4.0) | 15 (96.1) | 9 (10.0) |  |
| Per capital house income |  |  |  |  |  |  |
| Tertiles 1 | 79 (40.9) | 125 (42.1) | 65 (22.4) | 60 (22.0) | 22 (21.0) | <0.001 |
| Tertiles 2 | 75 (38.9) | 112 (37.7) | 113 (39.0) | 93 (34.1) | 20 (19.0) |  |
| Tertiles 3 | 39 (20.2) | 60 (20.2) | 112 (38.6) | 120 (44.0) | 63 (60.0) |  |
| Urbanization index |  |  |  |  |  |  |
| Tertiles 1 | 62 (31.6) | 115 (37.8) | 115 (38.9) | 110 (39.6) | 41 (38.3) | 0.545 |
| Tertiles 2 | 75 (38.3) | 96 (31.6) | 95 (32.1) | 95 (34.2) | 41 (38.3) |  |
| Tertiles 3 | 59 (30.1) | 93 (30.6) | 86 (29.1) | 73 (26.3) | 25 (23.4) |  |
| Region |  |  |  |  |  |  |
| Central | 52 (26.5) | 74 (24.3) | 72 (24.3) | 67 (24.1) | 27 (25.2) | 0.002 |
| Eastcost | 36 (18.4) | 57 (18.8) | 58 (19.6) | 56 (20.1) | 29 (27.1) |  |
| Northeastern | 50 (25.5) | 65 (21.4) | 46 (15.5) | 33 (11.9) | 10 (9.3) |  |
| Western | 58 (29.6) | 108 (35.5) | 120 (40.5) | 122 (43.9) | 41 (38.3) |  |
| **Sleep duration and blood pressure** | |  |  |  |  |  |
| Sleep duration (hours) | 9.33 (0.94) | 9.13 (0.87) | 9.07 (0.97) | 9.10 (0.90) | 8.49 (0.83) | <0.001 |
| Sleep duration (hours) |  |  |  |  |  |  |
| <9 | 36 (18.4) | 71 (23.4) | 80 (27.0) | 76 (27.3) | 64 (59.8) | <0.001 |
| ≥9 | 160 (81.6) | 233 (76.6) | 216 (73.0) | 202 (72.7) | 43 (40.2) |  |
| SBP (mmHg) | 94.57 (12.57) | 93.92 (11.23) | 98.20 (13.14) | 97.20 (12.10) | 103.11 (13.45) | <0.001 |
| DBP (mmHg) | 62.72 (9.65) | 62.32 (8.08) | 65.70 (8.94) | 63.94 (9.23) | 66.54 (10.15) | <0.001 |
| Elevated BP |  |  |  |  |  |  |
| No | 165 (84.2) | 275 (90.5) | 242 (81.8) | 235 (84.5) | 86 (80.4) | 0.022 |
| Yes | 31 (15.8) | 29 (9.5) | 54 (18.2) | 43 (15.5) | 21 (19.6) |  |

BMI: body mass index; SBP: systolic blood pressure; DBP: diastolic blood pressure; BP: blood pressure

**Table S2** Sample characteristics [(mean (SD) or n (percentage)) in girls over survey year from 2004 to 2015

|  | **2004 (n=178)** | **2006 (n=252)** | **2009 (n=234)** | **2011 (n=244)** | **2015 (n=102)** | **Time trend (*p*)** |
| --- | --- | --- | --- | --- | --- | --- |
| **Participants’ personal characteristics** | |  |  |  |  |  |
| Age(years) | 8.98 (1.45) | 9.98 (1.86) | 9.98 (1.88) | 9.71 (2.03) | 11.78 (0.82) | <0.001 |
| BMI z score | -0.10 (0.81) | -0.01 (0.97) | -0.05 (0.92) | 0.03 (1.05) | 0.23 (1.31) | 0.345 |
| Waist circumference | 56.06 (6.88) | 57.14 (7.25) | 58.42 (8.40) | 59.52 (11.13) | 65.15 (10.92) | <0.001 |
| Physical exercise frequency per week (times) | |  |  |  |  |  |
| <3 | 74 (46.5) | 50 (29.9) | 41 (24.0) | 53 (28.3) | 34 (40.5) | <0.001 |
| 3-7 | 66 (41.5) | 75 (44.9) | 76 (44.4) | 94 (50.3) | 39 (46.1) |  |
| ≥8 | 19 (11.9) | 42 (25.1) | 54 (31.6) | 40 (21.4) | 11 (13.1) |  |
| Screen time per day (hours) |  |  |  |  |  |  |
| <1 | 62 (39.2) | 77 (33.2) | 60 (26.0) | 56 (23.7) | 31 (33.7) | 0.001 |
| 1-2 | 61 (38.6) | 88 (37.9) | 88 (38.1) | 96 (40.7) | 22 (23.9) |  |
| ≥3 | 35 (22.2) | 67 (28.9) | 83 (35.9) | 84 (35.6) | 39 (42.4) |  |
| **Family and community information** | |  |  |  |  |  |
| Parents high BP history |  |  |  |  |  |  |
| No | 160 (97.0) | 227 (97.4) | 197 (93.8) | 195 (95.1) | 80 (94.1) | 0.310 |
| Yes | 5 (3.0) | 6 (2.6) | 13 (6.2) | 10 (4.9) | 5 (5.9) |  |
| Per capital house income (yuan) |  |  |  |  |  |  |
| Tertiles 1 | 86 (48.6) | 109 (44.1) | 82 (35.5) | 65 (27.2) | 20 (19.6) | <0.001 |
| Tertiles 2 | 56 (31.6) | 95 (38.5) | 79 (34.2) | 74 (31.0) | 23 (22.5) |  |
| Tertiles 3 | 35 (19.8) | 43 (17.4) | 70 (30.3) | 100 (41.8) | 59 (57.8) |  |
| Urbanization index |  |  |  |  |  |  |
| Tertiles 1 | 60 (33.7) | 88 (34.9) | 86 (36.8) | 87 (35.7) | 43 (42.2) | 0.790 |
| Tertiles 2 | 63 (35.4) | 90 (35.7) | 80 (34.2) | 74 (30.3) | 30 (29.4) |  |
| Tertiles 3 | 55 (30.9) | 74 (29.4) | 68 (29.1) | 83 (34.0) | 29 (28.4) |  |
| Region |  |  |  |  |  |  |
| Central | 38 (21.3) | 57 (22.6) | 62 (26.5) | 61 (25.0) | 15 (14.7) | <0.001 |
| Eastcost | 25 (14.0) | 38 (15.1) | 40 (17.1) | 50 (20.5) | 30 (29.4) |  |
| Northeastern | 48 (27.0) | 55 (21.8) | 27 (11.5) | 19 (7.8) | 8 (7.8) |  |
| Western | 67 (37.6) | 102 (40.5） | 105 (44.9) | 114 (46.7) | 49 (48.0) |  |
| **Sleep duration and blood pressure** |  |  |  |  |  |  |
| Sleep duration | 9.28 (0.91) | 9.13 (0.93) | 9.20 (0.93) | 9.16 (0.89) | 8.68 (1.03) | <0.001 |
| Sleep duration |  |  |  |  |  |  |
| <9h | 35 (19.7) | 65 (25.8) | 55 (23.5) | 52 (21.3) | 53 (52.0) | <0.001 |
| ≥9h | 143 (80.3) | 187 (74.2) | 179 (76.5) | 192 (78.7) | 49 (48.0) |  |
| SBP (mmHg) | 92.28 (12.10) | 93.65 (12.95) | 96.61 (12.63) | 95.54 (11.11) | 101.32 (11.60) | <0.001 |
| DBP (mmHg) | 60.76 (9.60) | 61.71 (8.94) | 64.90 (9.46) | 62.19 (8.56) | 65.73 (8.33) | <0.001 |
| Elevated BP |  |  |  |  |  |  |
| No | 157 (88.2) | 228 (90.5) | 193 (82.5) | 221 (90.6) | 91 (89.2) | 0.041 |
| Yes | 21 (11.8) | 24 (9.5) | 41 (17.5) | 23 (9.4) | 11 (10.8) |  |

BMI: body mass index; SBP: systolic blood pressure; DBP: diastolic blood pressure; BP: blood pressure


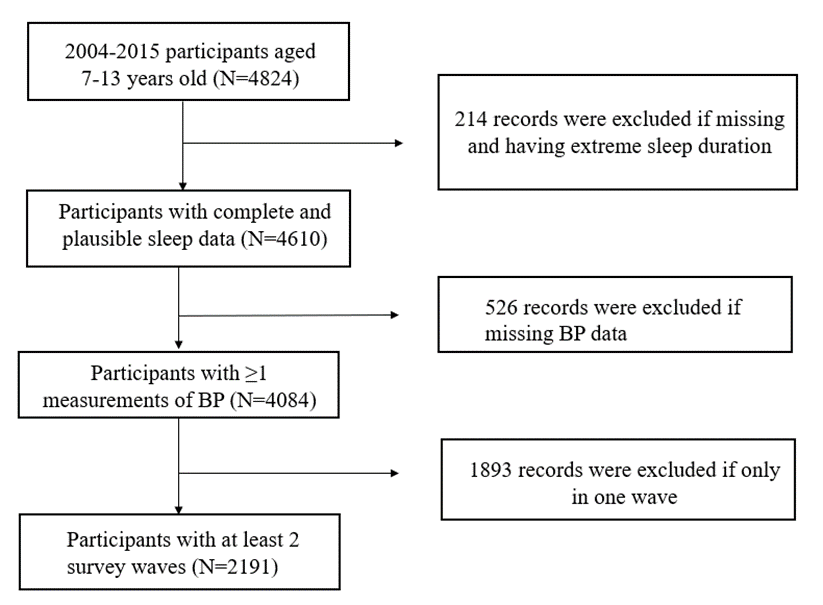


**Figure S1** Sampling flowchart and participants enrollment

**Table S3** The association of sleep duration with BP in 2004-2015.

|  | **SBP** | **P for interaction** | **DBP** | **P for interaction** | **Elevated BP** | **P for interaction** |
| --- | --- | --- | --- | --- | --- | --- |
|  | **β (95%CI)** |  | **β (95%CI)** |  | **OR (95%CI)** |  |
| **Crude** |  |  |  |  |  |  |
| continuous | -2.052(-2.619, -1.485) |  | -1.159(-1.561, -0.757) |  | 0.814 (0.708, 0.935) |  |
| <9h | **3.757 (2.582, 4.931)** |  | **1.964 (1.088, 2.840)** |  | **1.396 (1.066, 1.829)** |  |
| ≥9h | Reference |  | Reference |  | Reference |  |
| **Adjusted model^1^** |  |  |  |  |  |  |
| continuous | **-0.801(-1.416, -0.185)** |  | -0.388 (-0.823, 0.046) |  | **0.823 (0.704, 0.962)** |  |
| <9h | 1.098 (-0.165, 2.362) |  | 0.405 (-0.515, 1.324) |  | **1.361 (1.007, 1.841)** |  |
| ≥9h | Reference |  | Reference |  | Reference |  |
| **Adjusted model^2^** |  |  |  |  |  |  |
| continuous | -0.678 (-1.457, 0.101) |  | -0.210 (-0.748, 0.327) |  | 0.891 (0.731, 1.086) |  |
| <9h | 1.458 (-0.015, 2.931) |  | 0.641 (-0.433, 1.714) |  | 1.200 (0.837, 1.722) |  |
| ≥9h | Reference |  | Reference |  | Reference |  |
| **Interaction term** |  |  |  |  |  |  |
| Gender |  | 0.746 |  | **0.011** |  | 0.375 |
| BMI z score |  | 0.828 |  | 0.654 |  | 0.328 |
| WC |  | 0.290 |  | 0.905 |  | 0.808 |
| Physical activities |  | 0.058 |  | 0.454 |  | 0.568 |

OR, odds ratios; CI, confidence interval

Model 1 adjusted for age, gender and parents hypertension history

Model 2 additionally adjusted for survey time, waist circumference, BMI z score, urbanization, per capita household income, region, frequency of physical activities per week, screen time per day.

| **Table S4** The association of sleep duration with SBP, DBP, and elevated BP stratified by gender in 2004-2011. | | | |
| --- | --- | --- | --- |
|  | **SBP** | **DBP** | **Elevated BP** |
|  | **β (95%CI)** | **β (95%CI)** | **OR (95%CI)** |
| **Boy** | N=976 |  |  |
| Crude model |  |  |  |
| Continuous | **-1.698 (-2.565, -0.831) **** | -0.595 (-1.198, 0.008) | 0.845 (0.702, 1.016) |
| <9h | **2.415 (0.683, 4.147) **** | 0.447 (-0.842, 1.736) | 1.182 (0.803, 1.738) |
| ≥9h | Reference | Reference | Reference |
| Adjusted model^1^ |  |  |  |
| Continuous | -0.701 (-1.639, 0.237) | 0.119 (-0.510, 0.748) | 0.843 (0.682, 1.041) |
| <9h | 0.571 (-1.280, 2.423) | -0.780 (-2.067, 0.507) | 1.181 (0.771, 1.808) |
| ≥9h | Reference | Reference | Reference |
| Adjusted model^2^ |  |  |  |
| Continuous | -0.650 (-1.830, 0.530) | 0.554 (-0.236, 1.344) | 0.940 (0.724, 1.221) |
| <9h | 0.350 (-1.868, 2.567) | -1.367 (-2.859, 0.124) | 1.007 (0.595, 1.705) |
| ≥9h | Reference | Reference | Reference |
| **Girl** | n=815 |  |  |
| Crude model |  |  |  |
| Continuous | **-2.016 (-2.933, -1.098) ***** | **-1.857 (-2.505, -1.210) ***** | **0.676 (0.523, 0.874) **** |
| <9h | **3.745 (1.663, 5.828) ***** | **3.317 (1.732, 4.901) ***** | **1.994 (1.234, 3.223) **** |
| ≥9h | Reference | Reference | Reference |
| Adjusted model^1^ |  |  |  |
| Continuous | **-1.226 (-2.162, -0.291) **** | **-1.562 (-2.241, -0.883) ***** | **0.675 (0.507, 0.898) **** |
| <9h | 1.832 (-0.292, 3.956) | **2.543 (0.909, 4.177) **** | **2.017 (1.199, 3.394) **** |
| ≥9h | Reference | Reference | Reference |
| Adjusted model^2^ |  |  |  |
| Continuous | -0.831 (-1.970, 0.301) | **-1.336 (-2.145, -0.527) **** | 0.727 (0.501, 1.055) |
| <9h | 1.694 (-0.725, 4.113) | **2.193 (0.425, 3.962) *** | **1.909 (1.024, 3.556) *** |
| ≥9h | Reference | Reference | Reference |
| * P<0.05, ** P<0.01, * P<0.001.  OR, odds ratios; CI, confidence interval  Model 1 adjusted for age, parents’ hypertension history  Model 2 additionally adjusted for survey time, BMI z score, waist circumference, urbanization, per capita household income, region, frequency of physical activities per week, screen time per day. | | | |
